# Ear-Specific Hemispheric Asymmetry in Unilateral Deafness Revealed by Auditory Cortical Activity

**DOI:** 10.3389/fnins.2021.698718

**Published:** 2021-07-30

**Authors:** Ji-Hye Han, Jihyun Lee, Hyo-Jeong Lee

**Affiliations:** ^1^Laboratory of Brain & Cognitive Sciences for Convergence Medicine, Hallym University College of Medicine, Anyang-si, South Korea; ^2^Department of Otorhinolaryngology-Head and Neck Surgery, Hallym University College of Medicine, Chuncheon-si, South Korea

**Keywords:** unilateral deafness, hemispheric asymmetry, auditory spatial processing, sound localization, unilateral hearing loss (UHL)

## Abstract

Profound unilateral deafness reduces the ability to localize sounds achieved via binaural hearing. Furthermore, unilateral deafness promotes a substantial change in cortical processing to binaural stimulation, thereby leading to reorganization over the whole brain. Although distinct patterns in the hemispheric laterality depending on the side and duration of deafness have been suggested, the neurological mechanisms underlying the difference in relation to behavioral performance when detecting spatially varied cues remain unknown. To elucidate the mechanism, we compared N1/P2 auditory cortical activities and the pattern of hemispheric asymmetry of normal hearing, unilaterally deaf (UD), and simulated acute unilateral hearing loss groups while passively listening to speech sounds delivered from different locations under open free field condition. The behavioral performances of the participants concerning sound localization were measured by detecting sound sources in the azimuth plane. The results reveal a delayed reaction time in the right-sided UD (RUD) group for the sound localization task and prolonged P2 latency compared to the left-sided UD (LUD) group. Moreover, the RUD group showed adaptive cortical reorganization evidenced by increased responses in the hemisphere ipsilateral to the intact ear for individuals with better sound localization whereas left-sided unilateral deafness caused contralateral dominance in activity from the hearing ear. The brain dynamics of right-sided unilateral deafness indicate greater capability of adaptive change to compensate for impairment in spatial hearing. In addition, cortical N1 responses to spatially varied speech sounds in unilateral deaf people were inversely related to the duration of deafness in the area encompassing the right auditory cortex, indicating that early intervention would be needed to protect from maladaptation of the central auditory system following unilateral deafness.

## Introduction

Binaural hearing provides precise localization of sound sources while detecting them in the horizontal plane requires information regarding the temporal and sound level differences between the two ears. Adequate processing of these spatial cues also improves sound perception under adverse listening conditions since signal and background noise can be separated efficiently (Ellinger et al., 2017). However, hearing loss in one ear decreases the ability to process spatial cues properly, thereby yielding perceptual and communicative impairment (Chang et al., 2020). Perceptual issues that are mainly associated with unilateral hearing loss include sound localization and understanding speech in noisy environments (Bess and Tharpe, 1984; Rothpletz et al., 2012; Reeder et al., 2015; Firszt et al., 2017). For each task, the listener must successfully encode information on the interaural level difference (ILD), the interaural time difference (ITD), and monaural spectral cues (Shinn-Cunningham et al., 1998; Darwin, 2006; Schwartz et al., 2012). Unilateral hearing loss decreases the neural encoding of spatial and spectral information, which significantly impairs the localization of sound and understanding of speech-in-noise. Moreover, monaural hearing deprivation leads to maladaptive changes in the brain that cannot be recovered if deprivation occurs during a critical period of brain development (Harrison et al., 2005; Gordon and Kral, 2019). The findings from a previous report based on large datasets suggest that approximately 50% of children with unilateral hearing loss are faced with speech, language, and behavioral issues that cannot be accounted for by typical audiometric hearing tests (Bess and Tharpe, 1984; Lieu, 2004). This led to the assumption that perceptual impairment, linguistic elements, and perhaps the degree of neural plasticity together comprise the source of the issue (Kral et al., 2016).

Plasticity in the central auditory system following auditory deprivation induces functional and/or structural changes in the brain to reorganize neural networks (Moore et al., 1989; Fujiki et al., 1998; Ponton et al., 2001; Van der Haegen et al., 2016; Cartocci et al., 2019). Unilateral hearing loss develops a distinct pattern of brain reorganization that aims to compensate for poor peripheral representation of the spatial features of sound (Kral et al., 2013b; Keating et al., 2016). In normal hearing (NH) listeners, binaurally presented sounds are processed through the ipsilateral and contralateral auditory pathways, and at the cortex, they are predominantly processed in the hemisphere contralateral to the location of the sound source (McEvoy et al., 1993; Palomäki et al., 2005; Johnson and Hautus, 2010). Nonetheless, evidence supports the notion of differential processing of speech information in the brain. In the processing of speech signals, binaurally presented stimuli elicit stronger brain responses in the left hemisphere (Zatorre et al., 1992; Zatorre and Belin, 2001; Tervaniemi and Hugdahl, 2003). When considering the temporal and spectral information embedded in all speech sounds, the former show increased activation in the left area whereas the latter evoke opposing laterality (Schonwiesner et al., 2005; Okamoto et al., 2009). In unilaterally deaf (UD) people, frequently referred to as single-sided deafness, the normal pattern of hemispheric lateralization characterized by contralateral dominance for the stimulated ear decreases along with more bilateral activation over the two hemispheres compared to NH individuals (Bilecen et al., 2000; Khosla et al., 2003; Hine et al., 2008; Burton et al., 2012). In adult-onset unilateral deafness, the enhanced ipsilateral activity to the hearing ear can possibly be attributed to the increased bilateral activation rather than a decrease in the contralateral response (Vasama and Mäkelä, 1995; Scheffler et al., 1998; Ponton et al., 2001; Firszt et al., 2006; Li et al., 2006). Furthermore, in congenitally deafened children with electrical stimulation through a single cochlear implant (CI), the side and duration of deafness considerably influence the pattern and extent of cortical reorganization because accurate spatial processing requires binaural integration in the ascending auditory pathway that contains both the contralateral and ipsilateral projections from each ear (Henkin et al., 2008; Kral et al., 2013a).

Researchers have investigated differential ear effects on auditory cortical processing and deprivation-induced reorganization by applying neuroimaging and neurophysiology techniques (Khosla et al., 2003; Hine et al., 2008; Hanss et al., 2009; Burton et al., 2012; Maslin et al., 2013). Some studies have found that left-sided UD (LUD) adults exhibit extensive cortical reorganization, including increased bilateral activation or more ipsilateral activity on the side of stimulation (Khosla et al., 2003; Hanss et al., 2009). On the other hand, it has been recently reported that right-sided UD (RUD) people reveal strong activation in the frontal cortical regions not activated in LUD people. Furthermore, the increased frontal activation in the right-sided unilateral deafness is related to a higher level of listening effort or listening to degraded signals (Heggdal et al., 2019). Meanwhile, both left- and right-sided unilateral deafness increases the N1 dipole strength and shifts the dipole locations more medially (Maslin et al., 2013).

Taken together, the effect of the side of deafness on deafness-driven reorganization still remains unclear, with one possible explanation for the inconsistency being the nature of the stimuli applied to evoke brain responses. Several previous studies using non-complex stimuli such as tones (Bilecen et al., 2000; Pross et al., 2015), clicks (Khosla et al., 2003), and noise bursts (Burton et al., 2012; Firszt et al., 2013) have reported different results for the influence of the affected ear on the extent and pattern of brain reorganization. Considering that the listening paradigm and stimulus complexity can affect the way auditory information processes in the right and left hemispheres (Heggdal et al., 2019), a stimulation paradigm that is more perceptually relevant to deficits in the hearing function of UD people may shed light on the source of discrepancy. This speculation is more supported by the novel finding in UD children that irrespective of the deafness side, alpha and theta electroencephalography (EEG) activities were lateralized toward the side of the stimulation while they listened to speech-in-noise (Cartocci et al., 2019).

Given that scalp-recorded EEG represents neural mechanisms relevant to sound processing at different levels of the auditory system, it has been applied to assess the pattern and degree of cortical reorganization induced by monaural auditory deprivation. The general findings are that deafness in one ear results in substantial changes in neural activity from the subcortical to the central auditory system (Khosla et al., 2003; Hanss et al., 2009; Maslin et al., 2013). However, the findings from EEG studies on the mechanisms of cortical plasticity lack generalization, because interpretating the findings has been focused on assessing physiological changes *per se* rather than on relating the neural changes to behavioral perception. Defining a link between the brain and behavior is important to establish a biomarker for guidance on decision-making to provide hearing rehabilitation for the affected ear. This is particularly critical to children with asymmetric hearing loss since appropriate treatment can prevent deterioration of the cognitive/academic capacity that is often associated with unilateral hearing loss (Gordon et al., 2015).

Surprisingly, only a few researchers have attempted to relate the neurophysiological changes to behavioral performance in adults with unilateral deafness. For example, the findings from a recent study show a significant inverse relationship between speech perception ability, the duration of deafness, and cortical N1 responses in right-sided unilateral deafness, indicating that substantial neural plasticity occurs due to deafness in the right ear (Cañete et al., 2019). Similarly, a recent fMRI study also showed that a stronger dominance shift to the hemisphere ipsilateral to the better ear is significantly related to poorer horizontal sound localization in people with unilateral hearing loss (Vannson et al., 2020). Thus, the primary goal of this study is to determine whether electrophysiology can predict the behavioral perceptual ability of sound localization in UD adults. Another goal is to compare electrophysiological measures while passively listening to speech sounds in relation to the side of deafness.

In this study, we compared the cortical activities of long-lasting UD and NH participants with one ear noise-masked and occluded to simulate acute unilateral hearing loss. For this group, monaural occlusion caused temporal hearing deprivation and imbalance between the two ears. The experimental model allowed us to predict how unilateral hearing loss causes functional changes in the central nervous system at the initial stage of chronic unilateral deafness. Assuming that a developmentally critical period for brain plasticity induced by unilateral hearing loss exists (Popescu and Polley, 2010; Kral et al., 2013a), understanding the neural changes promoted by acute unilateral hearing loss would provide important insight into the optimal treatment for asymmetrical deafness. Evidence of cortical plasticity following acute unilateral hearing loss has been obtained from animal studies (Kamke et al., 2003; Kim et al., 2004; Eggermont, 2017). It has been reported that neurophysiological changes in the central auditory system are initiated soon after the loss of hearing sensation in one ear. Unilaterally deafened animals showed an immediate threshold shift in the hemisphere ipsilateral to the hearing ear with relatively normal activation in the contralateral side following monaural deprivation (Eggermont, 2017).

To date, only a limited number of studies directly examining the influence of acute unilateral hearing loss on the human brain have been conducted. The findings from these studies suggest that acute unilateral hearing loss can alter normally observed contralateral dominance for the stimulated ear and this unilaterally driven reorganization lasts for a year (Bilecen et al., 2000; Fan et al., 2015). In addition to the change in hemispheric lateralization, the findings from behavioral studies show an immediate adjustment in perceptual bias toward the hearing ear in horizontal localization (Slattery and Middlebrooks, 1994).

To examine the effects of the side and the duration of deafness on cortical reorganization, we compared N1/P2 cortical activities and the pattern of hemispheric asymmetry among chronic unilateral deafness, acute unilateral hearing loss, and NH controls. In addition, we carried out a behavioral sound localization test to separately correlate the measures of the cortical response and the hemispheric lateralization for right- and left-sided unilateral deafness. Based on previous literature suggesting differential effects of unilateral deafness on the cortical reorganization depending on the side of deafness (Ponton et al., 2001; Khosla et al., 2003; Hanss et al., 2009), we hypothesize that plastic changes in the brains of right- and left-sided deafness in chronic UD individuals affect cortical activity patterns and behavioral localization ability differently.

## Materials and Methods

### Participants

Ten adults who were RUD and 10 who were LUD were recruited. All UD participants were self-reported right-handed and had profound hearing loss in one ear (average pure-tone audiometry threshold > 90 dB HL from 0.25 to 4 kHz) without hearing devices for more than one year and NH (pure-tone thresholds < 20 dB HL from 0.25 to 4 kHz, and present otoacoustic emissions) in the other ear. Two subjects in the LUD group were congenitally deaf, and two from each of the LUD (age was five for both) and RUD groups (ages were six and eight) had childhood-onset deafness. In the RUD group, none of subjects was congenitally deaf. The etiology of unilateral deafness includes idiopathic, virus, Meniere’s disease, trauma, congenital, and auditory nerve deficiency. None of the UD groups had used a hearing aid before participating in this study. Thirty age- and gender-matched NH adults were recruited for comparison with the UD groups. The normal controls were sub-divided into three groups of 10: a NH group, 10 with their left ear noise-masked and occluded (LAUHL: left-sided acute unilateral hearing loss), and 10 with their right ears noise-masked and occluded (RAUHL: right-sided acute unilateral hearing loss). None of RAUHL, LAUHL, and NH group participants reported neurological and cognitive issues. Informed consent was obtained from all participants prior to testing (IRB no. 2019-02-019). A summary of the demographic data and statistical comparisons among the groups is provided in Table 1.

**TABLE 1:** Demographic data for the unilateral deafness, acute unilateral hearing loss, and normal hearing groups.

	LUD (*n* = 10)	RUD (*n* = 10)	RAUHL (*n* = 10)	LAUHL (*n* = 10)	NH (*n* = 10)	Statistics
Age (year, mean/SD)	41.9 ± 16.8	52.7 ± 6.2	44.1 ± 16.4	51.2 ± 8.3	52.2 ± 6.9	*F* = 1.78, *p* = 0.14
Gender (male/female)	4/6	4/6	3/7	3/7	3/7	*c*^2^ = 0.53, *p* = 0.97
Duration of deafness (year, mean/SD)	14 ± 16.2	19.6 ± 12.1				*t* = 0.86, *p* = 0.39
Deafness onset (year, mean/SD)	33.4 ± 22.4	24.7 ± 22.4				*t* = 0.27, *p* = 0.78
5 PTA (dB, mean/SD)	Lt ear: 101.0(15.9)	Lt ear: 11.0(4.3)	Lt ear: 17.5(6.4)	Lt ear: 16.7(6.0)	Lt ear: 11.0(8.3)	
	Rt ear: 7.6(6.2)	Rt ear: 99.4(15.5)	Rt ear: 16.9(6.3)	Rt ear: 17.1(5.8)	Rt ear: 7.2(5.7)	

*LUD, left-sided unilaterally deaf; RUD, right-sided unilaterally deaf; LAUHL, left-side acute unilateral hearing loss; RAUHL, right-side acute unilateral hearing loss; NH, normal hearing.*

### Stimuli

**FIGURE 1:**
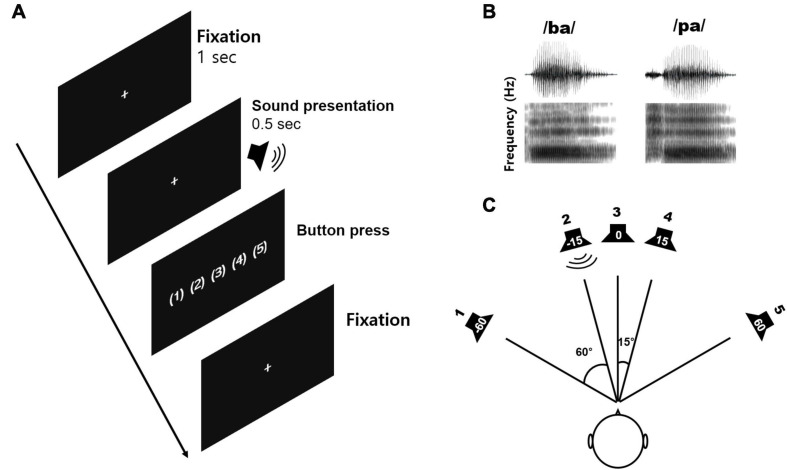
The sound localization paradigm and acoustic stimuli used in the study. **(A)** An example of the acoustic sequence for the active listening conditions. **(B)** The speech stimuli comprising/ba/and/pa/played for a duration of 0.5 s with varied inter-stimulus intervals. A new trial was started after the subject had performed a button press. **(C)** The sound localization paradigm including five different azimuth angles (–60°, –15°, 0°, +15°, and +60°).

Figure 1 shows the speech stimuli and sound localization paradigm applied in this study. Natural/ba/-/pa/speech stimuli were used to evoke cortical responses. The speech stimuli were recorded from utterances by a male speaker. The overall duration of each stimulus was 470 ms, and the voice onset times were 30 and 100 ms for/ba/and/pa/, respectively (Figure 1A). The stimuli were presented through a StimTracker (Cedrus Corporation, CA, United States) system that allowed for EEG synchronization with the sound and calibrated using a Brüel and Kjaer (2260 Investigator, Naerum, Denmark) sound level meter set for frequency and slow time weighting with a 1/2 inch free-field microphone.

Speech stimuli were presented through five loudspeakers at five different azimuth angles of −60, −15, 0, +15, and +60°, where ‘+’ indicates the right side while ‘−’ indicates the left side (Figure 1B). To evoke cortical responses, we used speech sounds presented from different horizontal locations since that allows the assessment of spectrotemporal processing closely related to neural sensitivity to spatial cues such as ITD and ILD (Bednar et al., 2017), and because the greater sensitivity to these spatial cues is positively related to behavioral performance while detecting sound locations (Palomäki et al., 2005).

### Procedure

All subjects participated in the tasks under passive and active listening conditions separately. For the passive listening condition, subjects were instructed to ignore any sounds while they watched a closed-captioned movie of their choice. For the active listening condition, participants were instructed to indicate location where a stimulus was presented via a button press. The attentive condition was always conducted first, followed by the passive condition. In this report, we present electrophysiological data under the passive listening only, and data from the active condition were used to obtain the behavioral performance of sound localization. For the active condition, each participant completed 10 familiarization trials of the procedure before undertaking the task. Prior to each block, participants were informed about the number of blocks and the upcoming task. Sound localization was measured for speech sounds at the five different azimuth angles mentioned above. For the active condition, stimuli were presented in 10 blocks of 1000 trials (200 trials for each of the five different azimuth angles), with each lasting 4 min, while the passive listening condition was presented across two blocks of 500 trials (200 trials for each of the azimuth angles) each lasting approximately 20 min. Breaks were given upon request. The total test time was approximately 1 h.

For the active listening conditions, the mean percent correct for the sound localization task was calculated as the number of correct sound location detections compared to all of them, while the reaction time was the average time taken for the subject to press the button to indicate the sound location. The reaction time was analyzed for all trials regardless of the correctness of localization, the only exception being missing button presses.

Subjects were seated in the center of the speaker array in a sound-attenuated booth. All of the speakers were located 1 m away from the subject at ear level and sounds were presented at 70 dB sound pressure level (SPL) for the NH and UD groups. We avoided roving the SPL level to keep the presentation level constant during the test. Note that for the acute UHL groups, one ear was covered with an earmuff and masked with a masking noise delivered through a Bluetooth earphone (Galaxy Buds^+^, Samsung, South Korea). The noise masker was speech-shaped noise taken from the speech stimuli used in this study with an overall intensity at a root-mean-squared (RMS) level of approximately 40 dB above the pure-tone threshold for each subject. To measure the level of the bluetooth earphone, the sound generated by the earphone was captured placing the sound level meter (Bruel & Kjaer 2250) at the end of physical ear canal (Aying et al., 2015). During the active condition, the inter-stimulus interval from sound offset to onset was varied since a new stimulus was only delivered when the subjects pressed the button, whereas the interval was fixed at 1.5 s for the passive condition. Prior to each trial, white fixation cross was displayed in the center of the black screen to minimize eye movements. In each experiment, the subjects were asked to fix their head positions at the center to minimize head movement that can affect both behavioral localization and EEG recording. All of the stimuli were randomly presented, and no performance feedback was given.

### EEG Recording

Electrophysiological data were collected using a 64-channel actiCHamp Brain Products recording system (Brain Products GmbH, Inc., Munich, Germany). An electrode cap was placed on the scalp with electrodes positioned at equidistant locations (Debener et al., 2005; Han and Dimitrijevic, 2015). The reference channel was positioned at the vertex while the ground electrode was located on the midline 50% of the distance to the nasion. Continuous data were digitized at 1,000 Hz and stored for offline analysis.

### Data Processing

Electrophysiological data were preprocessed using Brain Vision Analyzer 2.0 (Brain Products GmbH, Inc., Munich, Germany). Data were band-pass filtered (1–50 Hz) and down-sampled to 500 Hz. Visual inspection of the data included the removal of artifacts related to subject movement (exceeding 500 mV). Independent component analysis (ICA; Delorme and Makeig, 2004) implemented in the Brain Vision Analyzer was applied to remove artifacts related to eye blinking and movement, and cardiac activity.

After ICA artifact reduction, the data were low band-pass-filtered at 0.01–40 Hz and segmented from −200 to 1000 ms with 0 ms at the onset of the stimulus and re-referenced to the average reference. Averages were obtained for each of the azimuth angles. Subsequent peak detection was performed on the fronto-central electrodes for the N1/P2 components. Since we used an electrode cap with equidistant locations, N1/P2 were measured from the averaged activities of three electrodes at the Cz location in the international 10–20 system (Han and Dimitrijevic, 2015, 2020). N1 peaks were determined as the first negative potential between 80 and 150 ms after stimulus onset, while the most positive potential between 120 and 250 ms was defined as the P2 peak.

### Source Analysis

Averaged segments for each electrode location were analyzed in BESA (Brain Electrical Source Analysis). swLORETA was performed as has been previously described (Dimitrijevic et al., 2013; Han and Dimitrijevic, 2015). We chose swLORETA with two successive iterations because our previous studies using the analysis method have showed the most consistent N1 activations in the primary auditory cortex. swLORETA is a variation of sLORETA that includes depth weighting, and one of efficient methods to estimate brain source activation from scalp recorded potentials. For this analysis, we opted for an approach guided by mean area measurements of cortical waveform (Luck, 2014). The swLORETA analysis was conducted to obtain the time course of activation for N1. As a first step, swLORETA analysis yielded the maximal brain source activation as a function of time. For auditory N1 responses, swLORETA modeling was conducted in a 20 ms window in which maximal peaks were revealed in the grand mean waveform. In this step, two dipoles were inserted at each of the source maxima to obtain activation time courses. After source image files for subjects were obtained, we averaged all the individual image files for each experimental condition using a customized Matlab program. This averaged image file was considered the grand mean swLORETA source. The next step was to identify local maxima in the grand mean swLORETA source analysis outcome. Under most experimental conditions, the local maxima included the left and right auditory and frontal regions. Once the source maxima had been identified, the Talairach coordinates of the left and right auditory cortices were used to create grand averaged virtual source time (VST) activation for each condition. During this step, swLORETA was conducted to evaluate source activation of individual subjects in the time range from 0 to 500 ms. The swLORETA cortical activations at the previously determined Talariach coordinates (left and right auditory) were then extracted. In this step, the mean source activation in the 20 ms window was averaged to obtain VST activation separately for the left and right cortices. The VST was used to compute a lateralization index (LI) for each condition. Positive and negative LI values indicate right- and leftward asymmetries, respectively, and values exceeding ±0.2 were considered lateralized (Seghier, 2008).

### Statistical Analysis

For both behavioral sound localization and electrophysiology, repeated-measures analysis of variance (ANOVA) was performed to examine the effect of sound location and subject group on amplitudes and latencies for N1/P2 components as well as the percentage of correctly identifying sound locations. To test the hemispheric lateralization of the N1 source activity, we performed one-way ANOVA to compare the LIs among the subject groups for individual azimuths. *Post hoc* comparisons were conducted using Tukey’s Honest Significant Difference (HSD) test. To examine relationships between a behavioral measure and the hemispheric laterality in the UD groups, the LIs were each associated with the sound localization performances using Pearson product-moment correlations. Differences in the strength of the brain source space across the listening conditions were tested by applying paired *t*-tests corrected for multiple comparisons and Monte-Carlo resampling techniques implemented with BESA Statistics 2.0 (Maris and Oostenveld, 2007). Clusters of voxels with *p*-values of less than 0.05 were considered significant. BESA Statistics was also used to perform correlations between the duration of deafness and N1 source activity for each UD subject. This process yielded a correlation value for each voxel in the brain space related to the source activity and the audiologic factor. Non-parametric cluster permutation tests were conducted to determine the statistical significance of correlations between N1 source activation and the duration of deafness.

## Results

### Sound Localization

**FIGURE 2:**
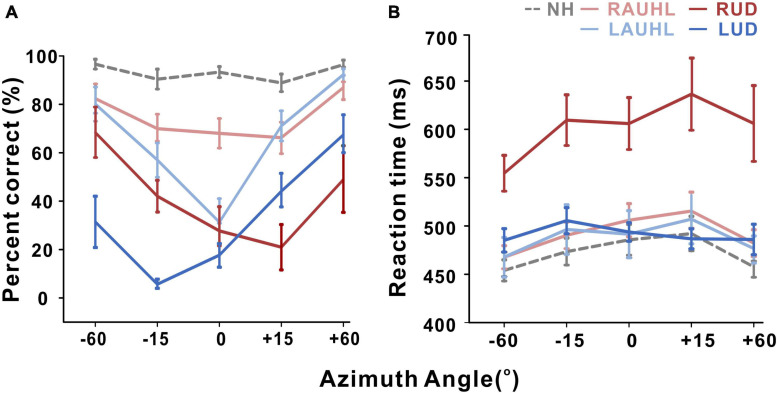
Mean percent correct score **(A)** and reaction time **(B)** as a function of azimuth angle for the sound localization task during the active listening condition. Error bars: the standard error of the mean. RUD, right-sided unilaterally deaf; LUD, left-sided unilaterally deaf; RAUHL, right-sided acute unilateral hearing loss; LAUHL, left-sided acute unilateral hearing loss; NH, normal hearing.

Although all of the subjects were able to complete the sound localization task during the active condition, data for one LUD subject were excluded from the statistical analysis since they were not reliable (he chose the same speaker for all of the trials). It should be noted that for both behavioral localization and EEG recording, monaural stimulation was provided to the UD groups while binaural input was supplied to the NH and AUHL groups. Averaged percent correct responses and reaction time as a function of azimuth during the active condition are shown in Figure 2. The averaged correct responses and reaction times for the groups across all of the azimuth angles and those for azimuth angles across all of the groups are shown in Figures 2A,B, respectively. A repeated-measures ANOVA was conducted to examine the effects of group (NH, RAUHL, LAUHL, RUD, and LUD) and azimuth angle (−60, −15, 0, +15, and +60°) on the sound localization task (Figure 2A). The percent correct responses revealed main effects for group [*F*(1,4) = 30; *p* < 0.001] and azimuth angle [*F*(4,16) = 26.9; *p* < 0.001]. Tukey’s HSD *post hoc* analysis revealed that the percent correct responses of the NH group were significantly higher than those of the RAUHL (*p* < 0.001), LAUHL (*p* = 0.003), RUD (*p* < 0.001), and LUD (*p* < 0.001) groups, while the RAUHL and LAUHL groups showed better performances compared to RUD and LUD groups (all *p* < 0.001). No significant differences were found between the RAUHL and LAUHL groups or between the RUD and LUD groups (all *p* > 0.05). The *post hoc* test results for the effect of azimuth angle on the percent correct responses showed that performances for 0° were lower than those for −60° (*p* < 0.001), + 15° (*p* < 0.004), and + 60° (*p* < 0.001) conditions. In addition, the percent correct responses for +15 and −15° were lower compared to −60 and +60° (all *p* < 0.001). For the reaction time in the sound localization test, repeated-measures ANOVA analysis revealed a main effect for group [*F*(1,4) = 9.66, *p* < 0.001], while a *post hoc* analysis showed that RUD had a significant delay when detecting sound locations compared to all other groups (all *p* < 0.001) (Figure 2B). No significant difference in the reaction time was found among the other groups (*p* > 0.05).

### N1/P2 Cortical Activity

#### Effects of Sound Location and Unilateral Deafness on N1/P2 Cortical Potentials

**FIGURE 3:**
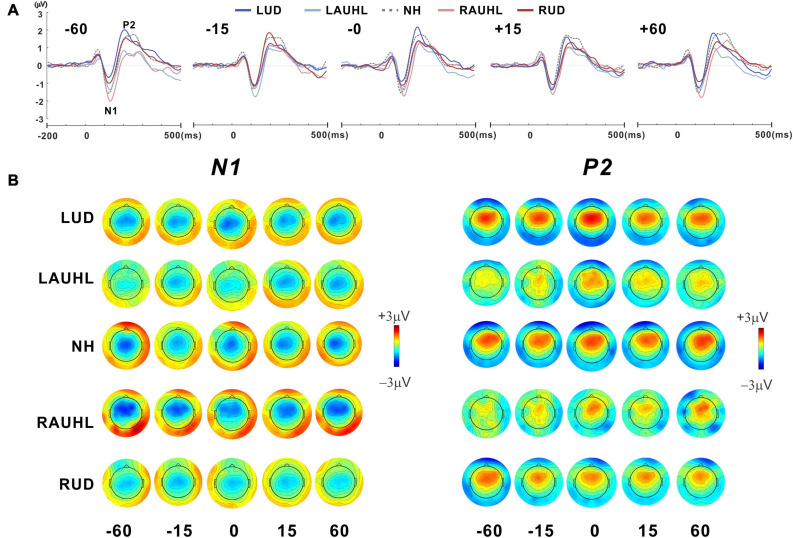
**(A)** Grand mean waveforms at five different azimuth angles (–60°, –15°, 0°, +15°, and +60°) for the subject groups recorded with fronto-central (FC) electrodes under the passive listening condition. **(B)** Topographical representation of the N1 and P2 responses as a function of azimuth angle for each subject group. RUD, right-sided unilaterally deaf; LUD, left-sided unilaterally deaf; RAUHL, right-sided acute unilateral hearing loss; LAUHL, left-sided acute unilateral hearing loss; NH, normal hearing.

Figure 3A shows the grand mean waveforms for stimuli at −60, −15, 0, +15, and +60° azimuth angles for the NH, RAUHL, LAUHL, RUD, and LUD groups. The overall response was characterized by an N1 evocation at around 100 ms after stimulus onset, followed by a P2 response. N1 peak modulations at the various azimuth angles were more apparent at −60° and less so at smaller ones. Corresponding topography plots for N1/P2 are shown in Figure 3B. Spatial distribution of the N1 topographies suggests that regardless of azimuth angle, the N1 activities in the RAUHL group were stronger than in the other groups. For the P2 responses, the topography suggests that the activities in the NH and LUD groups were larger than the RUD and acute unilateral hearing loss groups. Neither a change in brain activity nor hemispheric asymmetry as a function of azimuth was revealed by the plots.

**FIGURE 4:**
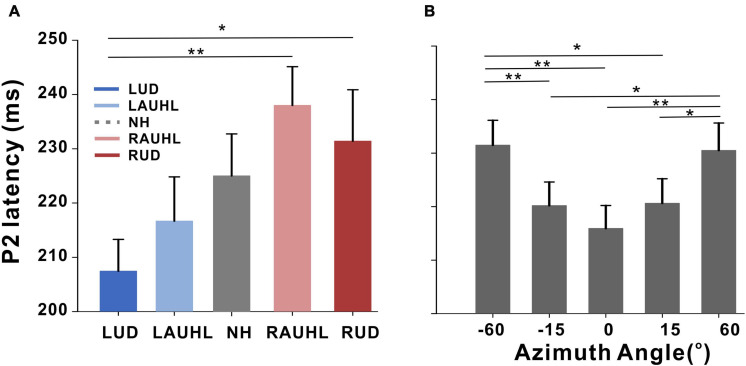
P2 latencies for the subject groups **(A)** and azimuth angles **(B)**. Error bars: the standard error of the mean. *P < 0.05, **P < 0.01. RUD, right-sided unilaterally deaf; LUD, left-sided unilaterally deaf; RAUHL, right-sided acute unilateral hearing loss; LAUHL, left-sided acute unilateral hearing loss; NH, normal hearing.

Repeated-measures ANOVA was applied to examine the effect of sound location and the group effect on N1/P2 measures, with which no significant differences for N1/P2 amplitudes were found. However, significant effects of group [*F*(_4_,_46_) = 3.05; *p* < 0.001] as well as sound location [*F*(_4_,_184_) = 5.37; *p* < 0.001] were found for latency measurements. As shown in the plot in Figure 4A, Tukey’s HSD *post hoc* test results show that the P2 latencies in the RAUHL and RUD groups were longer than those in the LUD group (*p* = 0.008 for RAUHL and *p* = 0.036 for RUD). For sound location, the P2 latencies at −60 and +60° were longer than at −15° (*p* = 0.006 and 0.013, respectively), 0° (*p* < 0.001 for both), and +15° (*p* = 0.009 and 0.017, respectively) (Figure 4B). No significant differences for group and sound location were found for N1 latency.

#### Differences in Source Space

**FIGURE 5:**
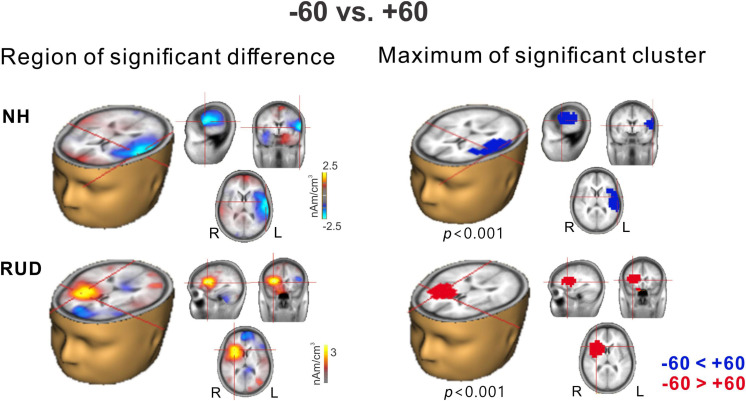
Cluster data representing significant differences between –60° and +60° azimuth angles in the brain source space. Blue indicates that +60° was greater than –60° (a negative difference) while red indicates that –60° was greater than +60° (a positive difference). Note that these clusters indicate which regions showed a significant difference while the crosshairs indicate a 3D point indicating the maximum difference between the azimuth angles. RUD, right-sided unilaterally deaf; NH, normal hearing.

Among the many possible comparisons of the conditions, we focused on −60° vs. +60° for the following reasons: (1) the results for the cortical potentials suggest that neural modulation as a function of sound location was more robust (Figure 3), and P2 latencies (Figure 4) were more delayed for ±60°compared to the other azimuth angles; (2) the findings in previous reports suggest that N1 cortical activity is larger for stimuli containing more prominent spatial cues than for less spatially distinguishable stimuli (Palomäki et al., 2005); and (3) given that the −60 and 60° azimuth angles are closer to the hearing and deafened ears than the other angles, these conditions could better represent the effect of unilateral deafness on source activation at the cortical level. Figure 5 shows *t*-test comparisons of −60 with +60° for the NH and RUD groups. For the NH group, comparing −60 and +60° revealed significant clusters (*p* = 0.001) that indicate greater contralateral activity for right-ear stimulation (+60°) in the left temporal area while passively listening to sounds from different locations (Figure 5 top). For the RUD group, a significant cluster (*p* < 0.001) in the right frontal lobe indicates larger contralateral activation to the hearing side (−60°) compared to the deaf side (+60°) (Figure 5 bottom). No significant differences were found for the RAUHL, LAUHL, and LUD groups.

#### swLORETA Source Analysis

**FIGURE 6:**
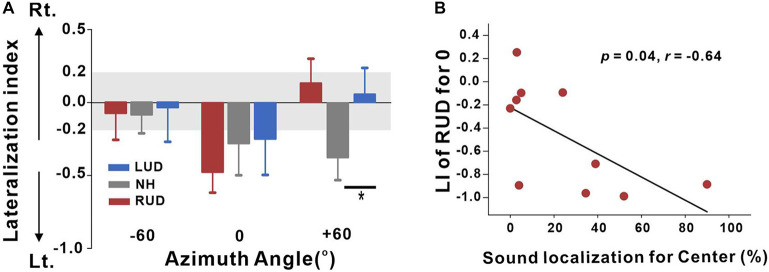
**(A)** Lateralization index (LI) plots at –60°, 0° (center), and +60° azimuth angles for the RUD, LUD, and NH groups. The gray regions in the LI plots indicate a 0.2 criterion for laterality. **(B)** Significant negative correlations for the RUD group between percent correct values on sound localization task and LIs for the center. Error bars: standard error of the mean. *P < 0.05. RUD, right-sided unilaterally deaf; LUD, left-sided unilaterally deaf; NH, normal hearing.

This analysis was conducted to measure cortical activation in the left and right auditory regions for each set of listening conditions. The VST activations were averaged for the left and right auditory areas to compute the LI. Given that significant differences in the source space analysis revealed when comparing +60 and −60°, we once again, focused on these conditions along with 0° to compare the hemispheric asymmetry among the LUD, RUD, and NH groups for each of the azimuth angles in terms of LI (Figure 6A). In general, the NH group revealed left hemispheric asymmetry when stimuli were presented from the center and right side, while leftward bias was stronger in response to right-side stimulation. In the LUD group, activity contralateral to the hearing side was increased for 0°, while no asymmetry was revealed at the other azimuth angles. Contrary to the LUD, the RUD group demonstrated strong ipsilateral activity to the hearing ear for 0°. One-way ANOVA analysis was conducted to examine group differences on the LI for each azimuth angle. The results indicate that the LIs for +60° significantly differed across the groups [*F*(_2_,_28_) = 3.59; *p* = 0.041], and a *post hoc* test showed that the N1 source activation in the NH group was lateralized to the left hemisphere whereas rightward asymmetry and no hemispheric bias were found for the RUD (*p* = 0.046) and LUD (*p* > 0.05) groups, respectively.

Analysis of the relationships between the LI and behavioral performance in the sound localization task was conducted separately for the LUD and RUD groups to examine whether differential cortical reorganization depending on the side of deafness is reflected in behavioral measures. The results in Figure 6B suggest that RUD showed more dynamic cortical reorganization in that the LIs in the RUD group for sound sources delivered to the center (0°) were significantly correlated with the sound localization performance (*r* = −0.64; *p* = 0.04). Moreover, asymmetry favoring the left hemisphere (ipsilateral to the hearing side) in the RUD group increased with better sound localization ability. No significant relationship was found in the LUD group (all *p* > 0.05).

#### N1 Source Relationship With Audiological Factors in Unilateral Deafness

**FIGURE 7:**
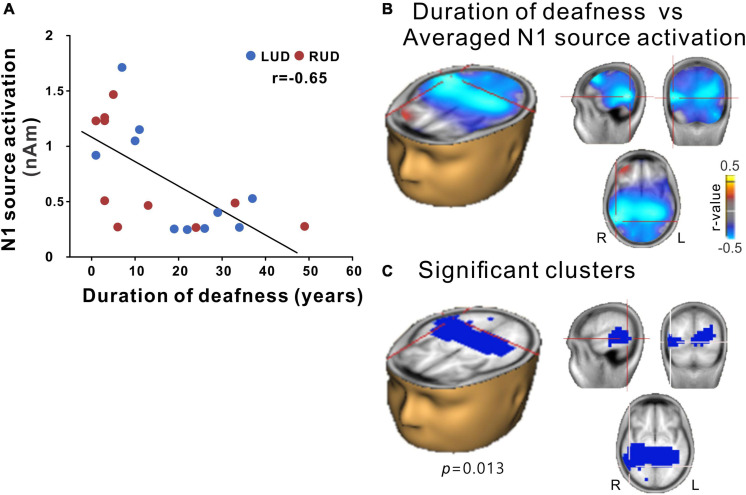
**(A)** N1 source activation correlation with the duration of deafness. **(B)** Voxels representing correlations between the duration of deafness and the averaged N1 source activation across the RUD and LUD groups. **(C)** Correlation data for voxels with peak correlations (crosshairs). Note that significant clusters were found in a broad region of the brain encompassing the bilateral temporal and occipital lobes with lateralization in the right hemisphere. RUD, right-sided unilaterally deaf; LUD, left-sided unilaterally deaf.

N1 source activation values were averaged across all azimuth angles to test them for correlation with the duration of deafness in the UD participants. The results in Figure 7 show a significant correlation between the averaged N1 source activation of all UD participants (including LUD and RUD) and the duration of deafness, indicating that lower N1 source activation was associated with a longer duration of deafness (Figure 7A). In the brain source space, a negative correlation (*r* = −0.65; *p* = 0.013) was found bilaterally (albeit more lateralized to the right hemisphere) in the temporo-occipital regions (Figure 7B). Figure 7C shows that significant clusters that survived after multiple comparison corrections were present in the right auditory cortex and right inferior temporal lobe.

## Discussion

The aim of this study was to characterize neurological changes and to relate these to behavioral sound localization in people with unilateral deafness. Cortical activities to speech sounds delivered at varied azimuth angles were compared between left- and right-sided unilateral deafness, and between acute and chronic unilateral hearing loss. The results of a source analysis indicate that N1 source activity in RUD individuals is lateralized ipsilateral to the hearing ear, while contralateral dominance from the hearing ear was found in LUD subjects for stimulation at the center. In addition, N1 activation in RUD people is lateralized ipsilaterally to the hearing side as their sound localization is better, thereby suggesting that an adaptive process in the auditory cortex in RUD individuals reorganizes the auditory pathway.

### Hemispheric Asymmetry in NH Individuals

Increased activity in the hemisphere contralateral to the perceived location of sounds during binaural stimulation has been reported previously (McEvoy et al., 1993; Soltani and Knight, 2000; Palomäki et al., 2005; Johnson and Hautus, 2010). Indeed, stronger activity in the contralateral hemisphere during right-side stimulation resembles laterality when processing spatial information (McEvoy et al., 1993). The contralaterality effects are associated with the neuroanatomical basis of functional lateralization in response to auditory stimulation that is characterized by larger neural responses in the contralateral auditory cortex (Scheffler et al., 1998; Jäncke et al., 2002). In the mammalian cortex, the majority of neurons tuning to spatial cues are more sensitive to contralateral input rather than ipsilateral or medial stimulation (Majkowski et al., 1971; Ahissar et al., 1992). In the current study, contrary to the right-side stimulation revealing the contralateral hemispheric lateralization, sounds from the left side produced no lateralization (Figure 6A). Along with the intrinsic limitation in the EEG measurements on spatial resolution, it could be due to increased activity of neurons ipsilateral to the side of stimulation rather than a reduced response by contralaterally selective neurons (Brancucci et al., 2004; Werner-Reiss and Groh, 2008). This can be accounted for by the channel model for sound localization that posits that auditory neurons for non-topographic rate coding are involved in two opposing channels broadly tuned to the left and right hemispheres used to tune spatial cues (Middlebrooks et al., 1994; Krumbholz et al., 2003; Magezi and Krumbholz, 2010). In addition to this concept, the results from later studies in which the model was adopted infer that along with contralateral channels for each hemisphere, there is an additional ipsilateral channel for the right hemisphere only. Furthermore, it has been suggested that the left auditory cortex is activated more strongly for sounds from the contralateral side than the ipsilateral one, while contralateral and ipsilateral stimulation are activated similarly in the right auditory cortex (Briley et al., 2013; McLaughlin et al., 2016). This notion was supported by the findings from lesion studies demonstrating that patients with left hemisphere damage were not able to locate sound sources from the contralateral hemispace only whereas those with right hemisphere damage revealed severely impaired sound localization from all locations (Clarke et al., 2000; Zatorre and Penhune, 2001; Spierer et al., 2009).

### Hemispheric Asymmetry in Unilateral Deafness

Unlike the NH participants, the functional lateralization of cortical activity in the brain of UD subjects in the current study was more ipsilateral to the hearing ear or more symmetrical between the two hemispheres. The alteration in hemispheric asymmetry in UD people during passive listening to sounds presented at different locations indicates that their auditory pathways for spatial processing had been substantially reorganized due to monaural hearing deprivation. The outcomes from several studies show that deafness in one ear causes a maladaptive change in the brain that can permanently disrupt the behavioral functioning of spatial hearing (Katz et al., 2015; Jiwani et al., 2016; Keating et al., 2016). Unilateral hearing loss that lasts during development weakens the neuronal representation of the impaired ear, which can affect the perception of relevant features for sound localization (Reeder et al., 2015; Easwar et al., 2017). In addition, the influence of unilateral hearing loss on cognitive function has shown to be greater in right-ear hearing-impaired children (Jensen et al., 1989). In the adult brain, the normal pattern of hemispheric asymmetry favoring the hemisphere contralateral to the side of stimulation is reduced in UD people (Bilecen et al., 2000; Khosla et al., 2003; Hine et al., 2008; Burton et al., 2012), and cortical reorganization is more extensive with earlier onset of unilateral hearing loss (Kral et al., 2013b; Gordon et al., 2015). Functional and anatomical evidence of the reorganization induced by asymmetrical hearing loss has been reported; neuroanatomical changes related to unilateral deafness include rearrangement of neuronal connectivity (Moore et al., 1989), a reduction of spatially tuned neurons (Hancock et al., 2010, 2013), and increased inactive neuronal sites with lower firing rates (Popescu and Polley, 2010; Tillein et al., 2016) in both the subcortical and cortical levels of the auditory system.

In the current study, hemispheric asymmetry in response to speech sounds differed depending on the side of deafness such that N1 responses of RUD people are lateralized to the hemisphere ipsilateral to the intact ear, whereas LUD people revealed contralateral bias in response to stimuli at the center (Figure 6A). Furthermore, the RUD group showed a significant reversal of hemispheric dominance from the right hemisphere to the left hemisphere that was most probably in those who showed better behavioral sound localization (Figure 6B). Given that the auditory system can change the way the brain processes spatial information when adapting to monaural hearing to compensate for decreased spatial sensitivity (Kral et al., 2013b; Keating et al., 2016), higher activity in the hemisphere ipsilateral to the hearing ear in the better performers reflects the process of neural adaptation for recovery from asymmetric hearing loss that is achieved by strengthening the ipsilateral auditory pathway.

Interestingly, right- and left-sided deafness caused different patterns of hemispheric asymmetry while behavioral sound localization in UD subjects significantly decreased regardless of the side of deafness. The discrepancy between the behavioral and EEG data could have occurred because of different routes of sound processing for the active and passive listening tasks. In this study, we evoked cortical responses during passive listening whereas participants actively listened to sounds to detect their locations for the behavioral test. Behaviorally, sound localization is biased toward the side with the intact ear following unilateral deafness (Slattery and Middlebrooks, 1994). In contrast, auditory stimulation with spatial cues provokes ear-specific patterns of hemispheric laterality in unilateral deafness that could be influenced by the role of the right auditory cortex for spatial processing in unilaterally deafened people. It has been shown that hemispheric selectivity predisposes the right hemisphere to process sound location in both hearing and deafened subjects (Palomäki et al., 2005; Johnson and Hautus, 2010). Furthermore, the lateralized cortical responses are stronger during active compared to passive listening due to top-down processing (Fine et al., 2005).

When comparing source activities in response to sounds at −60 and +60°, greater frontal activation was revealed in the right-sided deaf participants whereas the source activities remained located in the auditory area of the NH subjects. Similar anterior activation in RUD individuals was observed by Heggdal et al. (2019) during listening to degraded signals, which was interpreted as the recruitment of the frontal region for higher-order cognitive processing to detect sound location (Li et al., 2019). The contrast in activation between −60 and +60° also shows that no laterality was observed for left-sided deafness. Symmetrical cortical activation over the auditory cortices only occurred in left-sided deafness that is related to the cortical organization induced by unilateral deafness (Hanss et al., 2009).

Contrary to our findings, those from previous studies assessing differential ear effects on hemispheric lateralization suggest that left-sided unilateral deafness incurs more extensive cortical reorganization (Khosla et al., 2003; Hanss et al., 2009). The authors found significant reversal of lateralization favoring the hemisphere ipsilateral to the intact ear or a more symmetrical activation pattern over the auditory cortex in left-sided unilateral deafness. Furthermore, functional reorganization due to left-sided deafness was observed in a larger area of the brain compared to right-sided unilateral deafness (Burton et al., 2012). The discordance in hemispheric asymmetry between our study and previous findings could be due to the listening paradigm used to evoke neural responses. In the present study, cortical activity was elicited by natural speech sounds varied by azimuth angle to reflect auditory processing of spatial information, while most previous studies evoked auditory responses using artificially modulated tones (Bilecen et al., 2000; Li et al., 2006; Burton et al., 2012; Pross et al., 2015), clicks (Khosla et al., 2003), or noise bursts (Firszt et al., 2013). Given that auditory N1/P2 responses are more sensitive to rapidly changing spatial cues than other responses, they reflect the asymmetrical processing of the auditory system for auditory spatial information more efficiently (Kaiser and Lutzenberger, 2001; Palomäki et al., 2005; Krumbholz et al., 2007). In line with this assumption, a recent study using speech stimuli demonstrated that right-sided unilateral deafness increases cortical activation in the frontal region of the brain, while no noticeable change in activity was revealed in left-sided unilateral deafness (Heggdal et al., 2019). Thus, we suggest that spatially varied speech stimuli produce distinct azimuth-angle-specific lateralization in LUD and RUD people.

### P2 Latency Associated With the Side of Deafness and the Location of the Sound Source

P2 latency to speech stimuli varied by azimuth angle was longer in the RUD and RAUHL subjects (Figure 4A), which reflects the greater cognitive effort required for cortical processing of speech and spatial cues in right-sided unilateral deafness (Tong et al., 2009; Rao et al., 2010). The longer P2 latencies in the RUD and RAUHL groups could be related to the increased reaction time for sound localization revealed by our behavioral data. It has been shown that the spatial processing of sounds is predominantly processed in the right hemisphere (Kaiser et al., 2000). Considering that the contralateral auditory pathway is stronger than the ipsilateral one during monaural stimulation, a longer processing time is required for individuals with right-sided hearing loss. The auditory P2 response represents the neural mechanisms relevant to neuroplastic changes in the higher level of the auditory system related to auditory discrimination (Tremblay and Ross, 2007), memory (Näätänen and Picton, 1987), attention (Alain et al., 2010), and learning (Lee J. et al., 2020). This is relevant to the locations of the P2 responses that include the planum temporale accommodated in the anterior auditory cortex and the auditory association cortices (Crowley and Colrain, 2004). In the current study, the P2 response was evoked by stimuli presented from different locations during passive listening, which is associated with implicit attention being given to discriminate the sound location. It is known that the cortical P2 response contains endogenous characteristics associated with cognitive processing, such as attention (Hillyard et al., 1973; Woldorff and Hillyard, 1991). Analogous to the mismatch negativity, the P2 response is modulated by inattentive auditory processing for auditory discrimination even during passive listening. Therefore, we consider that the prolonged P2 response in right-sided unilateral deafness could reflect the attention-related physiological changes due to top-down modulation for the passive auditory discrimination of speech stimuli (/ba/vs./pa/) or different sound locations. Indeed, right-side specific P2-response prolongation is possibly related to strong cortical reorganization revealed by exposure to our source data.

Meanwhile, a more prolonged P2 response was prevalent at larger azimuth angles (−60 and +60°) than smaller ones (−15, +15, and 0°) (Figure 4B). The longer latency for increasing azimuth angle is related to modulation of the cortical response by changes in spatial features. Specifically, greater changes in the ITD and the interaural phase or coherence elicited a delayed cortical response due to a longer processing time (Sonnadara et al., 2006; Picton, 2013). Using the mismatch negativity evoked by infrequent changes in sound location, Sonnadara et al. (2006) found a longer latency in the positive peak in response to a large angle (90°) compared to a smaller one; they also alluded that this can be partially interpreted by applying the spatial channel theory suggested by Boehnke and Phillips (1999) who posited that 0 and 30° are processed in the same spatial channel whereas 0 and 90° (angles larger than 30°) are not. Moreover, according to the theory, spatial location information belonging to different channels is not processed in the lower level of the auditory system but rather in the higher order auditory cortex (Vannson et al., 2020). In this sense, the prolonged latency at the greater azimuth angles could be associated with the precise spatial processing properties of the central auditory system for between-channel discrimination.

### The Relationship Between N1 Source Activity and Duration of Deafness

In the current study, source-level N1 activities were significantly associated with the duration of deafness in the area encompassing the temporal-occipital regions. This result suggests that the longer the duration of unilateral deafness, the more substantial neurological changes at the cortical level. These findings are in agreement with those from previous studies in that the changes in cortical activity in UD individuals occur gradually over time after the onset of deafness (Bilecen et al., 2000; Ponton et al., 2001; Cañete et al., 2019). In UD adults, a decrease in N1 response is associated with reduced ability in speech-in-noise perception, and the N1 activity contralateral to the side of the stimulation weakens as the duration of deafness becomes longer (Cartocci et al., 2019; Cañete et al., 2019). Greater neurological changes with longer hearing deprivation could be related to the notion that early-onset unilateral deafness incurs a detrimental effect on the representation of spatial information at both the cortical and subcortical levels (Hancock et al., 2010, 2013). It is recognized that neural processes for sound localization at the brainstem and cortical levels are well-coordinated through the ascending and descending pathways, while the neural representation of spatial features is improved by callosal connections between two hemispheres (Hausmann et al., 2005; Krumbholz et al., 2007). However, in unilateral deafness, the deafness-driven reorganization of neural mechanisms for spatial information at the brainstem and later stages of processing in the auditory cortex presumably reduces the neural activity for sound processing, and the longer duration of deafness could further exacerbate the neural changes. A finding from studies in children with CIs supports this assumption because children with simultaneous bilateral implantation demonstrate better sensitivity to ITD cues than those with sequential cochlear implantation, which is possibly due to the longer period of unilateral deafness (Gordon et al., 2015; Easwar et al., 2017). Results from previous studies also suggest that unilateral deafness reduces behavioral sensitivity to spatial cues as well as the neural encoding of spatial features of sounds more severely in people with a longer duration of hearing loss (Hancock et al., 2013; Firszt et al., 2015), which may not fully recover even after treatment (Lee H.J. et al., 2020).

Significant clusters were found in the areas of the temporal and occipital lobes. Recent neuroimaging studies investigating cross-modal plasticity have revealed evidence that the deafness in one ear could reorganize broad areas of the brain, including the frontal, temporal, and occipital regions (Shang et al., 2019). In fact, cross-modal plasticity has been studied extensively in bilaterally deafened people since the absence of auditory function requires substantial reorganization of the brain to compensate deprived sense (Lee et al., 2001; Fine et al., 2005; Sandmann et al., 2012). In bilateral CI patients, structural and functional plasticity occurs in the visual cortex (Smittenaar et al., 2016; Han et al., 2019). Meanwhile, similar to what has been observed in CI users, functional reorganization involving the auditory and visual cortices could be possible in unilateral deafened individuals. A future study is needed to examine the cross-modal takeover of auditory-visual stimulation in unilateral deafness.

### Cortical Plasticity in People With Acute Unilateral Hearing Loss

In our study, horizontal sound localization in subjects with acute unilateral hearing loss was behaviorally poorer than in NH participants but better than in chronic UD people. Specifically, the accuracy of sound localization decreased drastically in the LAUHL group for auditory stimulation in the center position. Given that the spatial cues required for normal sound localization are known to be processed in the right hemisphere (Kaiser et al., 2000), temporarily induced monaural stimulation decreased localization accuracy much more severely in the LAUHL subjects. In addition, behavioral sound localization decreased by approximately 20% for all azimuth angles in both simulated unilateral hearing loss groups. Unlike our findings, ear-plugging one ear of ferrets reduces the localization accuracy more severely (by almost 60%) (Nodal et al., 2012). The lesser effect of occluding one ear in humans on sound localization could be related to the effects of training and/or spectral-shaped cues of the pinna of the hearing ear that improves the detection capability of sound location with monaural hearing (Van Wanrooij and Van Opstal, 2004). Firszt et al. (2015) showed the efficacy of sound localization training in unilaterally deafened people using monosyllabic words and spectral/temporal sounds on a spectrogram, thereby suggesting the clinical need for localization training of these individuals.

Neurologically, P2 responses were prolonged in individuals with RAUHL, while the normal pattern of contralateral lateralization to the hearing side decreased (shown in Supplementary Figures 1, 2). Several researchers have attempted to measure neurological and behavioral changes induced by occluding one ear to examine the extent and speed of recovery after the occlusion, as well as the underlying neural plasticity (Slattery and Middlebrooks, 1994; Keating et al., 2016). Similar results to our finding have been reported in that monoaurally earplugged listeners respond with more bias toward the hearing side (Butler et al., 1990; Slattery and Middlebrooks, 1994) or displace the location within the compressed range in the auditory space due to reduced sensitivity to spatial cues (Oldfield and Parker, 1986). In humans, neuroplasticity incurred by the temporary loss of hearing is evident at the subcortex level such that acute unilateral hearing loss reduces threshold levels in the auditory reflex (Brotherton et al., 2016). Similarly, findings from animal studies also reveal substantial changes in neuronal spatial tuning for sound localization in the auditory primary cortex following the occlusion of one ear. In earplugged adult ferrets, the accuracy of behavioral localization was reduced by monaural occlusion (Nodal et al., 2012). Furthermore, deactivating the auditory cortex of the ferrets resulted in a drastic performance deficit, indicating that neural changes in the auditory cortex play a role in deafness-driven reorganization. Therefore, the acute onset of binaural imbalance not only incurs the abnormal perception of spatial cues for sound localization but also causes neurological changes both in the subcortex and the high level of the auditory cortex.

### A Lack of Deafness and Sound Location Effects on N1 Amplitude

Although the differences did not reach a significant level, the N1 amplitude change as a function of azimuth angle was more prominent at larger angles than smaller ones. The pattern of cortical response in adults was characterized by greater activity in response to more spatially distinctive stimuli (McEvoy et al., 1993; Johnson and Hautus, 2010). Larger amplitudes and longer latencies of the N1 response to a stimulus contralateral to each hemisphere have also been reported (McEvoy et al., 1993; Palomäki et al., 2005). One possibility for no difference in N1 response is using binaural stimulation to evoke it. Previous studies have shown that monaural stimulation elicits larger N1 responses than presenting sounds binaurally (Reite et al., 1981; McEvoy et al., 1993). Moreover, contralateralized processing of spatial cues does not occur in response to binaural stimulation (Jäncke et al., 2002; Zimmer et al., 2005). Decreased contralaterality is related to the ascending auditory pathway that includes both ipsi- and contralateral connections to the auditory cortex. There is evidence that the contralateral pathway contains a larger number of neurons and faster transmission speed compared to the ipsilateral one (Majkowski et al., 1971). For a direct comparison between monaural and binaural stimulation, a future study needs to be conducted to explore cortical activity in response to monaurally presented stimuli.

### Limitation of the Current Study

In this study, we used speech stimuli that are known to be predominantly processed in the left hemisphere. Assuming that sounds are mainly processed in the contralateral hemisphere, auditory inputs are transferred via the corpus callosum in right-sided deafened individuals, which yielded prolonged P2 latency and behavioral delay for the horizontal localization task. To exclude the potential stimulus effect, non-speech stimuli should be applied to evoke brain responses.

Topographical representation of the N1 and P2 amplitudes indicates noticeable group differences in that they are stronger in left-sided deafness than right-sided deafness. However, there were no significant differences in the amplitude measurements between the groups. Further study with a larger sample size and different statistical methods would perhaps yield more meaningful results.

In this study, we presented acoustic stimuli at a constant level to unilateral deaf subjects that allow level cues due to the head shadow effect. However, it would be better to apply randomly roved sound levels to assess sound localization in the listening condition that the UD subjects face, because it can reduce azimuth-related head shadow cues. Moreover, it would allow us to examine the behavioral localization when only spectral cues were available. There is strong evidence that listeners are more dependent on the monaural spectral cues than the sound level and sound source location cues for azimuth localization when binaural inputs are not available due to monaural plugging (Van Wanrooij and Van Opstal, 2007). This could be relevant to better performances at ±60° compared to the other angles in the UD subject, which revealed in our study. This is further supported by the previous literature that the information to resolve perceptual uncertainty in ILD and ITD cues is spectral in nature (Carlile et al., 2005). Nonetheless, the contribution of the spectral information to sound localization in the UD subjects is known to be different depending on the individual differences in the localization ability. In a localization test using randomly roved sound levels, all UD subjects heavily depend on the head-shadow cues, while only better UD performers are able to use the spectral cues (Van Wanrooij and Van Opstal, 2004). A future study applying randomized listening condition would therefore to help better understand the use of different sound localization cues in the UD individuals and the underlying neural mechanisms.

## Conclusion

In summary, we provided new information that right-sided unilateral deafness incurs greater deafness-driven reorganization compared to left-sided unilateral deafness, as evidenced by stronger activity ipsilateral to the hearing ear. This notion is further supported by the finding that contralateral hemispheric lateralization of RUD people shifts toward the ipsilateral hemisphere with better behavioral localization, suggesting that neural adaptive changes strengthen the ipsilateral auditory pathway to compensate for decreased spatial sensitivity. In addition, simulated acute unilateral hearing loss decreased the behavioral localization accuracy as well as the normal contralateral dominance for spatial processing. Finally, neuroplasticity in the auditory cortex of UD adults is more prominent in people with a longer duration of monaural deprivation, indicating that early intervention for unilateral deafness may change the degree of unilaterally driven reorganization that is closely linked to the spatial sensitivity for sound localization.

Our results signifying substantial neuroplasticity in regions of the auditory cortex of unilaterally deafened people indicate that early intervention is needed to protect from maladaptive reorganization caused by asymmetric input. Intervention can include (re)activating the deaf ear using appropriate hearing-assistive devices such as a bone-anchored hearing aid or CI for profound hearing loss (Dorman et al., 2016; Laszig et al., 2017; Mertens et al., 2018). It has also been claimed that restoration of binaural hearing with a CI actually improves the speech-in-noise and sound localization capabilities of unilateral deaf individuals regardless of the deafened side (Ehrmann-mueller et al., 2020).

## Data Availability Statement

The raw data supporting the conclusions of this article will be made available by the authors, without undue reservation.

## Ethics Statement

The studies involving human participants were reviewed and approved by Institutional Review Board at the Hallym University College of Medicine. The patients/participants provided their written informed consent to participate in this study.

## Author Contributions

J-HH and H-JL contributed to the conception and design of the study. J-HH and JL collected and analyzed the data. All authors contributed to writing the manuscript.

## Conflict of Interest

The authors declare that the research was conducted in the absence of any commercial or financial relationships that could be construed as a potential conflict of interest.

## Publisher’s Note

All claims expressed in this article are solely those of the authors and do not necessarily represent those of their affiliated organizations, or those of the publisher, the editors and the reviewers. Any product that may be evaluated in this article, or claim that may be made by its manufacturer, is not guaranteed or endorsed by the publisher.
