# Corrigendum: Ear-Specific Hemispheric Asymmetry in Unilateral Deafness Revealed by Auditory Cortical Activity

**DOI:** 10.3389/fnins.2021.793365

**Published:** 2021-11-03

**Authors:** Ji-Hye Han, Jihyun Lee, Hyo-Jeong Lee

**Affiliations:** ^1^Laboratory of Brain & Cognitive Sciences for Convergence Medicine, Hallym University College of Medicine, Anyang-si, South Korea; ^2^Department of Otorhinolaryngology-Head and Neck Surgery, Hallym University College of Medicine, Chuncheon-si, South Korea

**Keywords:** unilateral deafness, hemispheric asymmetry, auditory spatial processing, sound localization, unilateral hearing loss (UHL)

In the original article, there was a mistake in Figure 6. In the figure, there were inconsistencies when comparing left and right panels. In the left panel (Figure 6A), the left laterality was indicated by negative values (-); however, it was positive in the right panel (Figure 6B). Since we have decided to indicate the left hemispheric laterality by the negative values, a relationship between the laterality index and sound localization in right-sided deafness should be inversed, meaning a negative correlation. The corrected Figure 6 appears below.

**Figure 6:**
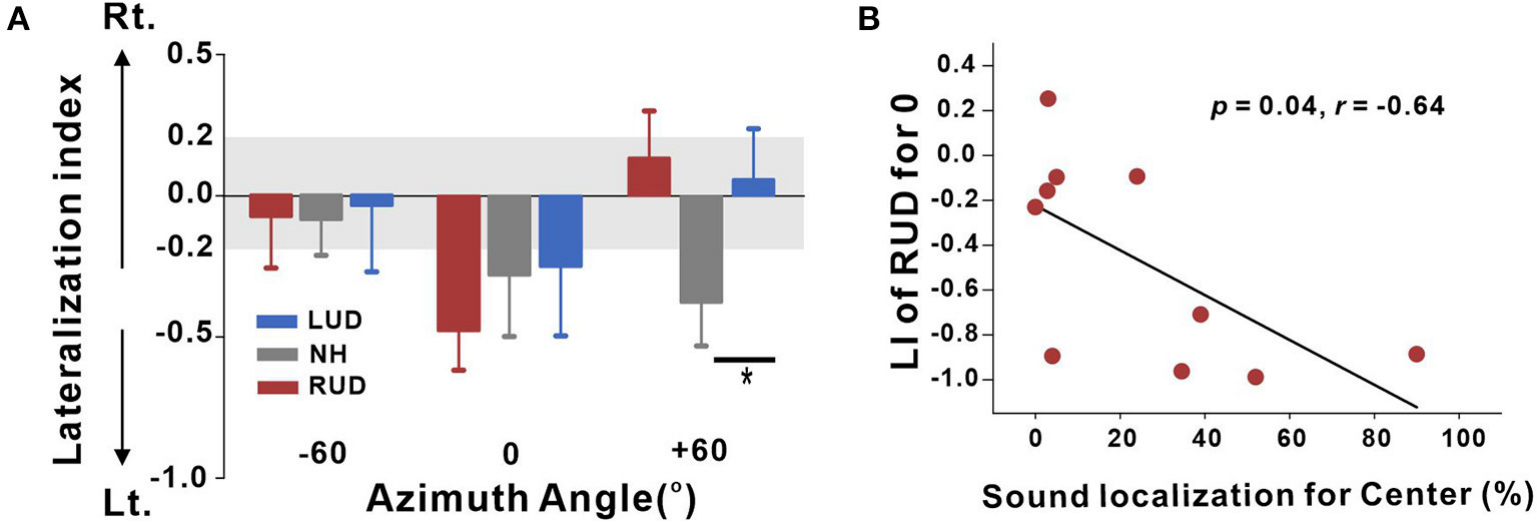
**(A)** Lateralization index (LI) plots at −60°, 0° (center), and +60° azimuth angles for the RUD, LUD, and NH groups. The gray regions in the LI plots indicate a 0.2 criterion for laterality. **(B)** Significant negative correlations for the RUD group between percent correct values on sound localization task and LIs for the center. Error bars: standard error of the mean. ^*^P < 0.05. RUD, right-sided unilaterally deaf; LUD, left-sided unilaterally deaf; NH, normal hearing.

In the original article, there was a mistake in the legend for Figure 6 as published. The relationship should be changed from positive to negative correlation. The correct legend appears below.

In the original article, there was an error. The r value for the correlation between sound localization and LI of RUD should be negative (-) rather than positive value.

A correction has been made to *Results*, subsection *swLORETA Source Analysis*, Paragraph 2:

Analysis of the relationships between the LI and behavioral performance in the sound localization task was conducted separately for the LUD and RUD groups to examine whether differential cortical reorganization depending on the side of deafness is reflected in behavioral measures. The results in Figure 6B suggest that RUD showed more dynamic cortical reorganization in that the LIs in the RUD group for sound sources delivered to the center (0°) were significantly correlated with the sound localization performance (*r* = −0.64; *p* = 0.04). Moreover, asymmetry favoring the left hemisphere (ipsilateral to the hearing side) in the RUD group increased with better sound localization ability. No significant relationship was found in the LUD group (all *p* > 0.05).

The authors apologize for these errors and state that this does not change the scientific conclusions of the article in any way. The original article has been updated.

## Publisher's Note

All claims expressed in this article are solely those of the authors and do not necessarily represent those of their affiliated organizations, or those of the publisher, the editors and the reviewers. Any product that may be evaluated in this article, or claim that may be made by its manufacturer, is not guaranteed or endorsed by the publisher.

