# Supplementary material for: Ear-Specific Hemispheric Asymmetry in Unilateral Deafness Revealed by Auditory Cortical Activity
Source: Front Neurosci. 2021 Jul 30;15:698718. doi: 10.3389/fnins.2021.698718 (PMC8363420; doi:10.3389/fnins.2021.698718)
Supplement: Supplementary file 1 [file Data_Sheet_1.PDF]

Supplement table 1. Mean N1 and P2 amplitudes and latencies.

| Group | Azimuth (mean/SD) | N1 Amplitude | N1 Latency | P2 Amplitude | P2 Latency |
|---|---|---|---|---|---|
| RUD | -60 | -1.5 (0.63) | 126 (20.69) | 2.01 (0.98) | 238.6 (35.47) |
| | -15 | -1.65 (0.6) | 122.6 (18.06) | 1.76 (0.92) | 228.6 (35.92) |
| | -0 | -1.63 (0.87) | 122.2 (18.79) | 1.84 (0.62) | 229.8 (38.41) |
| | +15 | -1.49 (0.83) | 121.8 (22.9) | 1.69 (1.09) | 226.2 (28.95) |
| | +60 | -1.31 (1.01) | 121.8 (18.24) | 1.65 (1.01) | 233.8 (38.8) |
| LUD | -60 | -1.27 (1.46) | 119.8 (17.52) | 2.35 (1.29) | 215 (26.25) |
| | -15 | -1.51 (1.45) | 123.2 (13.4) | 2.19 (1.03) | 201.6 (16) |
| | -0 | -1.15 (1.41) | 122.2 (15.67) | 2.55 (1.02) | 207.2 (20.89) |
| | +15 | -1.6 (1.61) | 121.6 (12.53) | 2.05 (0.81) | 204.4 (26.47) |
| | +60 | -1.51 (1.58) | 120.8 (12.62) | 2.27 (1.01) | 209 (29.33) |
| RAUHL | -60 | -2.44 (1.45) | 132.4 (17.45) | 1.25 (1.1) | 245.8 (32.92) |
| | -15 | -2.22 (1.25) | 132.8 (20.61) | 1.41 (0.97) | 217.6 (26.64) |
| | -0 | -2.34 (1.26) | 132.2 (19.33) | 1.59 (0.9) | 224.4 (35.53) |
| | +15 | -2.11 (1.25) | 129.6 (20.15) | 1.44 (1.01) | 231.4 (36.12) |
| | +60 | -2.21 (1.12) | 132.8 (17.91) | 1.72 (1.14) | 270.8 (9.98) |
| LAUHL | -60 | -1.53 (0.99) | 124 (9.97) | 1.41 (0.85) | 222.8 (34.7) |
| | -15 | -1.43 (0.75) | 131 (14.24) | 1.64 (1.08) | 222.6 (32.37) |
| | -0 | -1.62 (0.87) | 126.2 (11.17) | 1.68 (1.16) | 217.2 (27.31) |
| | +15 | -1.5 (0.83) | 125.6 (10.31) | 1.44 (1.12) | 218.4 (32.06) |
| | +60 | -1.55 (1.07) | 126.4 (14.5) | 1.37 (1.21) | 202.4 (23.09) |
| NH | -60 | -1.85 (1.08) | 119.8 (11) | 2.06 (0.77) | 234.9 (32.9) |
| | -15 | -1.57 (0.98) | 118.9 (12.75) | 1.95 (0.99) | 229.63 (37.9) |
| | -0 | -1.86 (1.16) | 113.63 (11.37) | 2.11 (0.96) | 202.4 (28.64) |
| | +15 | -1.69 (1.39) | 116.18 (9.93) | 2.18 (1.04) | 222.54(38.27) |
| | +60 | -1.82 (1.11) | 119.2 (11.94) | 2.3 (1.02) | 236 (32.4) |
