# Supplementary figures and images for: Ear-Specific Hemispheric Asymmetry in Unilateral Deafness Revealed by Auditory Cortical Activity
Source: Front Neurosci. 2021 Jul 30;15:698718. doi: 10.3389/fnins.2021.698718 (PMC8363420; doi:10.3389/fnins.2021.698718)

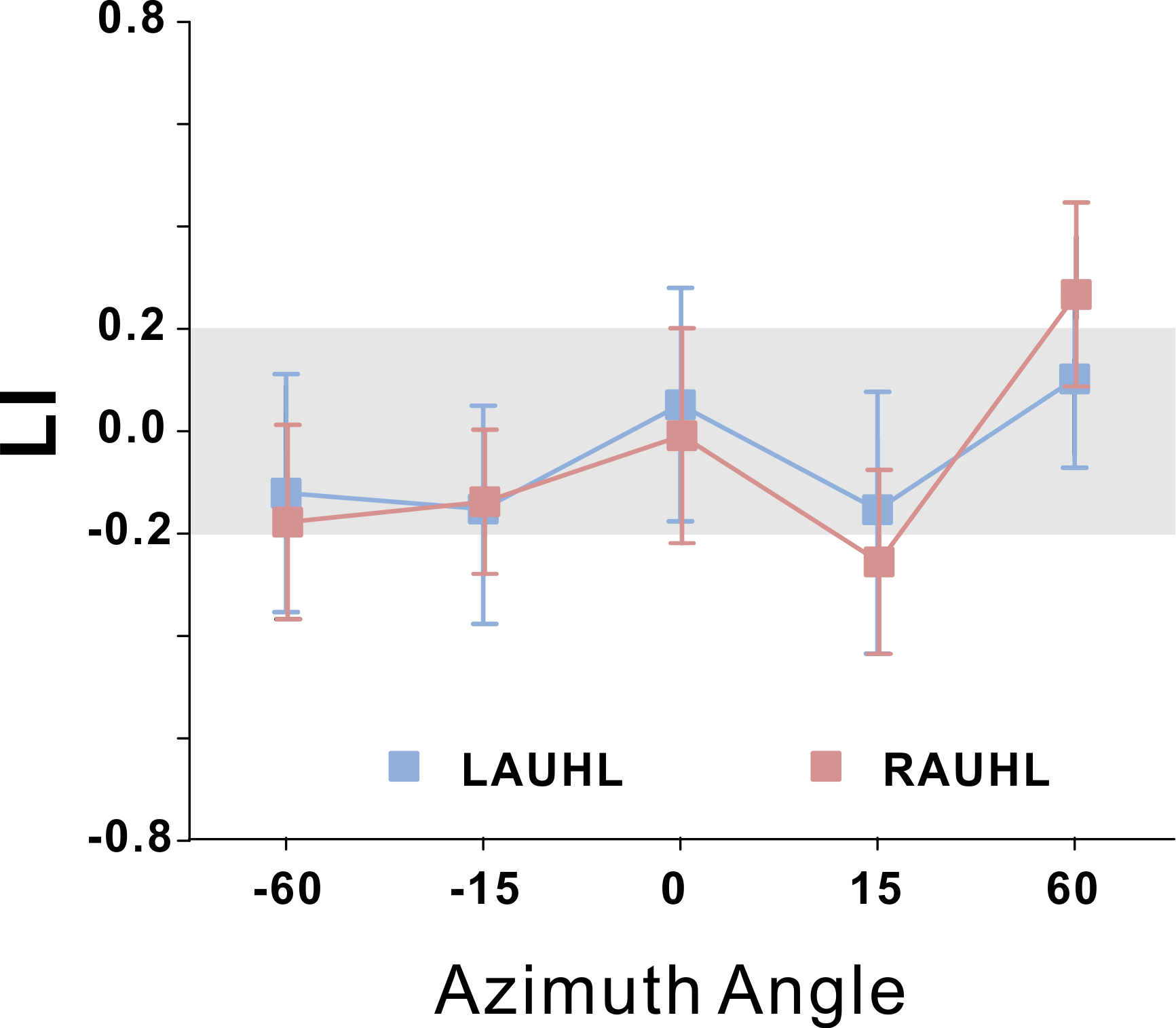

Supplement: Supplementary file 2 [file Image_1.TIF]

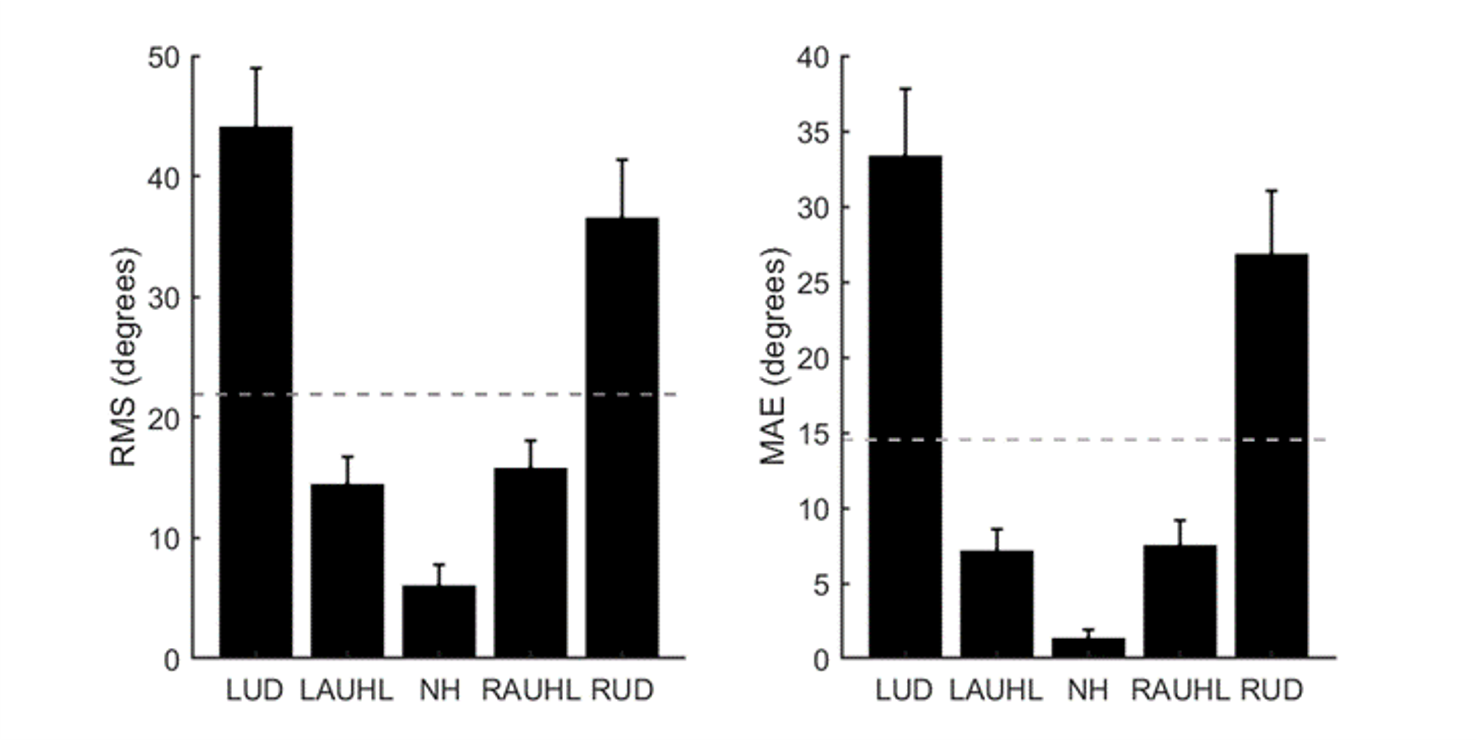

Supplement: Supplementary file 3 [file Image_2.TIF]
